# The Painful Tweet: Text, Sentiment, and Community Structure Analyses of Tweets Pertaining to Pain

**DOI:** 10.2196/jmir.3769

**Published:** 2015-04-02

**Authors:** Patrick J Tighe, Ryan C Goldsmith, Michael Gravenstein, H Russell Bernard, Roger B Fillingim

**Affiliations:** ^1^University of Florida College of MedicineDepartment of AnesthesiologyGainesville, FLUnited States; ^2^University of FloridaDepartment of AnthropologyGainesville, FLUnited States; ^3^University of FloridaDepartment of Community Dentistry and Behavioral ScienceGainesville, FLUnited States

**Keywords:** Twitter messaging, emotions, text mining, social networks

## Abstract

**Background:**

Despite the widespread popularity of social media, little is known about the extent or context of pain-related posts by users of those media.

**Objective:**

The aim was to examine the type, context, and dissemination of pain-related tweets.

**Methods:**

We used content analysis of pain-related tweets from 50 cities to unobtrusively explore the meanings and patterns of communications about pain. Content was examined by location and time of day, as well as within the context of online social networks.

**Results:**

The most common terms published in conjunction with the term “pain” included feel (n=1504), don’t (n=702), and love (n=649). The proportion of tweets with positive sentiment ranged from 13% in Manila to 56% in Los Angeles, CA, with a median of 29% across cities. Temporally, the proportion of tweets with positive sentiment ranged from 24% at 1600 to 38% at 2400, with a median of 32%. The Twitter-based social networks pertaining to pain exhibited greater sparsity and lower connectedness than did those social networks pertaining to common terms such as apple, Manchester United, and Obama. The number of word clusters in proportion to node count was greater for emotion terms such as tired (0.45), happy (0.43), and sad (0.4) when compared with objective terms such as apple (0.26), Manchester United (0.14), and Obama (0.25).

**Conclusions:**

Taken together, our results suggest that pain-related tweets carry special characteristics reflecting unique content and their communication among tweeters. Further work will explore how geopolitical events and seasonal changes affect tweeters’ perceptions of pain and how such perceptions may affect therapies for pain.

## Introduction

Twitter is the most popular microblogging website in the world, with more than 1 billion tweets posted every 3 days [1]. The Twitter application programming interface (API) permits researchers to search for keywords in content that is posted in short, 140-character “tweets” written from a variety of fixed locations and mobile computing platforms, thus offering insights into the day-to-day discourse of personal and geopolitical events [2-5]. This API also captures information pertaining to “retweets” and “mentions,” whereby a Twitter user specifically tags a tweet to another individual, which permits the tracking of Twitter communities. These unique characteristics of Twitter have spurred investigations of tweets on topics ranging from politics, finance, and sports to health-related issues, such as H1N1 influenza epidemiology, tobacco cessation, and disaster response [2,3,6-12]. To the best of our knowledge, there have been no investigations into how pain has been discussed across Twitter.

For many, pain represents a ubiquitous yet thankfully transient experience in everyday life. However, for more than 100 million Americans, an otherwise transient experience fails to subside, progressing into chronic pain conditions costing upwards of US $635 billion dollars. More than half of hospitalized patients and 50%-75% of cancer patients die while suffering from moderate to severe pain. In the acute pain setting, more than 60% of surgical patients suffer from moderate to severe pain following surgery [14,15]. Depending on the type of surgery, up to 50% of patients will progress directly to a chronic pain condition stemming from their surgery [14,15]. A wealth of evidence points to many specific psychosocial factors that modulate pain intensity. Given the strong emotive content of social media postings, it seems prudent to investigate how pain is discussed across widespread social media platforms such as Twitter.

Two core domains of Twitter content exploration are (1) content analysis or the extraction of meaning from the tweet itself and (2) community structure analysis or the measurement of social networks based on patterns of retweets among tweeters. Content analysis includes simple measurement of word use and association along with quantification of tweet affect via sentiment analysis [16-21]. Using rules and statistical modeling techniques developed on manually annotated corpora, or body, of texts, tweets can be classified as positive in sentiment (eg, “No pain no gain, great workout, I love exercise! ”) or negative in sentiment (eg, “Ouch, my back really hurts, so sad I will miss soccer practice, bummer! ”) [4,16,17,22-29]. Content analysis can offer insight into how tweeters incorporate the term “pain” into their daily tweets, measuring the concepts discussed and the emotional tags implicit within such tweets.

Community structure analyses of retweets measure the connectedness of Twitter-based social networks. Prior observations suggest that chronic pain may be associated with, or even induce, different forms of social isolation [30-36]. Contrary to that, pain itself may be a focus of commiseration as a pointed topic of discussion, such that psychosocial therapies often focus on improving social support systems and/or minimizing social isolation [37,38]. Community structure analyses of retweet patterns for pain-related tweets can help determine whether online communications about pain reflect a more limited, intimate network of communiqués versus a more expansive dissemination of pain-related content.

To the best of our knowledge, such analytic approaches have yet to be applied to tweeters’ communiqués pertaining to pain. Here, we explore the content of more than 65,000 tweets from around the world, each containing the term “pain.” We discuss the possibility that using a combination of text analysis and network analysis, Twitter can be leveraged to study the qualitative, multidimensional facets of pain unobtrusively in the context of daily living. We had 2 objectives: (1) to evaluate the context and sentiment of pain-related tweets and (2) to compare the connectedness of Twitter-based social networks pertaining to pain against those networks containing cross-culturally universal emotion terms (happy, excitement, sad, fear, tired, anguish) and a sample of common objective terms (apple, Manchester United, Obama) [39,40]. Hypotheses pertaining to these objectives were as follows:

1. We hypothesized the existence of specific topics associated with pain; in the null, a random set of terms associated with pain.
2. We hypothesized a mixture of positive and negative sentiment in pain-related tweets; in the null, a uniformly negative sentiment.
3. We hypothesized a unique connectivity pattern of Twitter-based retweet networks pertaining to pain when compared with networks pertaining to other emotive and nonemotive terms; in the null, a connectivity pattern indistinguishable from other Twitter-based retweet networks.

## Methods

### Overview

The institutional review board at the University of Florida (IRB-02) declared this project exempt as a survey study on public data. Two series of analyses were conducted. The first examined the content of tweets related to pain and the second explored the social networks of tweeters retweeting content related to pain. Each set of analyses employed a separate corpus of pain-related tweets.

### Content Analysis of Tweets

#### Overview

In classical content analysis, human readers identify the themes or concepts in a set of texts. We used automated, computer-based content analysis to extract the concepts mentioned frequently in 65,000 tweets pertaining to pain. This approach to extracting concepts from social media platforms has previously been demonstrated by a number of teams addressing a wide range of issues [12,23,26,41-46]. We also analyzed the context of pain-related words to distinguish between positive and negative uses of those words. Here, we describe our approach to the content analysis of pain-related tweets with a special emphasis on the quantification of the sentiment used within these pain-related tweets. Additional technical details are available in Multimedia Appendix 1.

#### Pain Tweet Corpus Generation

Data were collected during a single search in September of 2012. We first created a function to request 1500 of the most recent English-language tweets containing the term “pain” [9]. The date and time of posting of each tweet were collected. The time of day documented for each tweet was then adjusted to reflect the local hour of time for the city in question when the tweet was posted. To identify the city of origin for each tweet, the function searched for tweets posted from within a 100-mile radius of the latitude and longitude specified for a set of 50 large, English-speaking cities from around the world. Although this approach provides a geolocation for each tweet, it is important to recognize that this approach did not capture nongeolocated tweets, potentially biasing the results toward those individuals with more sophisticated tweeting devices that were able to provide geolocation capabilities via Global Positioning System (GPS) and/or cellular location methods [47-50]. The United States was oversampled to provide a suitable basis for exploratory correlations between city demographic and climatic data with pain-related tweet sentiment. Additional technical details are available in Multimedia Appendix 1.

The search was repeated for each of the 50 selected cities. A total of 10% of the tweets from each city were visually inspected for quality assurance. Data from one city were found to be corrupt, we believe, due to an error in our query code and were removed from further analysis. Given that all other tweets were collected in a batch search, we elected not to repeat the collection of this city’s data given concerns for skewing of sampling due to different search times.

Of note, tweets in this analysis were not specifically searched for “#pain,” whereby the hashtag is used as a metatag to mark a tweet as containing a specific topic [18]. We opted to search for “pain” as a general search term to discern how the term was used in the normal discourse of daily life. A search specifically for “#pain” would have returned only those tweets wherein the tweet’s topic of interest was identified by the tweeter as pain, thus biasing the returned context and sentiment of the tweet contents.

Tweets obtained from this sample were consolidated into a pain tweet corpus, consisting of the text of all collected tweets. Here, “corpus” (and its plural, “corpora”) refers to a body of texts on which analyses are conducted.

#### Term-Term Association Measurements With Graph Analysis

To measure how often terms in a tweet were associated with the term “pain” or other terms, we used an analytical approach known as graph analysis [6]. Each term was represented as a node in a network and the relationships between terms were the links, or edges, connecting those nodes. Note that in the content-analysis experiments, nodes represented individual words and communities represented groups of associated words connected by links or edges. Whenever 2 terms were found in the same tweet, those 2 terms were considered to share a link. The linkage of nodes by edges lends itself to quantitative analysis via those matrix algebra methods that underpin graph theory. Additional technical details are available in Multimedia Appendix 1.

For each term, the total degree centrality was first calculated by counting how many different links, or edges, that term had to other terms within the corpus. By examining how well groups of terms were associated with one another, but not other terms or groups of terms, communities of terms commonly associated with one another were determined using a community detection algorithm based on the Louvain method [3].

#### Sentiment Analysis

Sentiment scoring of tweets combined a rule-based approach with a statistical modeling system to create a hybrid sentiment classifier [51]. The rule-based approach used the AFINN (named for the author, Finn Årup Nielsen) listing of weighed positive and negative keywords [52]. The AFINN wordlist is a list of manually labeled English terms that have been rated for positive versus negative polarity, which has been explicitly validated for use in microblog environments such as Twitter. This was supplemented with emoticon terminology to enhance the accuracy of the rule-based classifier [53-55]. Additionally, the rule-based approach incorporated negation terms and contractions within 5 terms of a positive or negative keyword to reverse the sentiment to a score of ±1. By summing the positive and negative weights of keywords identified within a given tweet, the polarity (positive versus negative sentiment) could be calculated along with a confidence level. The statistical model employed a Naïve Bayes algorithm with a smoothed relative frequency for text normalization and a feature-ranking algorithm based on the risk ratio [48]. Additional technical details are available in Multimedia Appendix 1.

Classifier scores were compared with human ratings of sentiment using an interrater agreement scoring system. Given initial concerns over the implementation of sentiment analysis, each reviewer was engaged in a short didactic session by the principal investigator (PT) and given specific examples, including “Exercise was great! No pain, no gain!” for positive sentiment versus “Twisted ankle, pain unbearable, so sad to miss game!” for negative sentiment. However, given the subjective nature of sentiment analysis and exploratory nature of this characterization, more formal training was not offered. Given the historically poor interannotator agreement with sentiment analysis, some have suggested that the decidedly deterministic results provided by rule-based and classifier-based sentiment analyses may offer methodological advantages over those offered by human annotators [11,56,57].

Exploratory analyses correlated elementary demographic and climatic data for US cities with the proportion of pain-related tweets with positive sentiment for those cities. This exploratory analysis was motivated by historical clinical wisdom as well as work by Keller et al [58] and Jamison et al [59] that suggests an association between cooler climates, decreased mood, and greater pain intensity. Population, population density, median age, percentage of high school graduates, percentage with bachelor’s degree or higher, median household income, and number of individuals below poverty level were obtained from the 2010 US Census [60]. Given its rural nature, data for Phoenix Township, Arkansas, were extracted from data pertaining to Pope County, Arkansas, in the absence of specific data from the US Census. Percentages of individuals without health insurance were extracted from the 2010 Small Area Health Insurance Estimates dataset on a per-county basis [61]. Climate data for the month of September for each city were aggregated from the 1981-2010 normals published by the National Oceanic and Atmospheric Administration [62], and included average high temperatures and average number of precipitation days with greater than 0.01 inches of rain. Climate data for Phoenix Township, Arkansas, was adapted from the Little Rock, Arkansas, climate area.

### Community Structure of Twitter-Based Social Networks Related to Pain

#### Collection of Retweet Data

In March of 2013, we searched Twitter for the following terms: pain, #pain, happy, excitement, sad, fear, tired, anguish, apple, Manchester United, and Obama [63]. Emotional terms were selected as samples of positive and negative pain-related terms from a prior compilation of 15 universally applicable, cross-cultural emotional affects [39,40]. Comparator terms were empirically chosen to reflect discourse on common topics in an effort to compare against topics with widespread media attention across different public domains following discussion with coauthors. Each search was filtered for English-language tweets and was limited to 1500 returned tweets by the Twitter API. Additional technical details are available in Multimedia Appendix 1.

#### Description of Social Network Analysis Measurements

After import into Gephi, the network- and node-level metrics were calculated for each search term [8,64,65]. Network-level metrics included node and edge count, network diameter, average path length, density, and the number of weakly and strongly connected components [66,67]. Calculated node-level metrics included the number of modularity communities, the total degree centrality, in-degree centrality, and out-degree centrality [3,65].

To determine how often other emotion terms were tweeted by those individuals engaged within the pain retweet network, we sampled 100 individuals from the pain term network who submitted a tweet containing the term “pain” as a retweet or mention to another Twitter user. Using the userTimeline function (a specific piece of computer code within the twitteR package created for use with the R programming language) in the twitteR package, we then requested up to the last 100 tweets for each of these individuals. The text of these tweets was combined into a corpus. This corpus was then searched for the number of occurrences of each of the 6 emotion terms (happy, excitement, sad, fear, tired, anguish) and 3 objective terms (apple, Manchester United, Obama) previously noted. For each term, its frequency and its frequency in proportion to the frequency of the term “pain” were calculated and reported. Additional technical details are available in Multimedia Appendix 1.

## Results

### Content of Pain-Related Tweets

#### Graph Analysis

Analyses were conducted on a version of the pain tweet corpus in which identical tweets were removed; this is referred to as the reduced pain tweet corpus. For the graph analysis, the reduced pain tween corpus contained 47,958 nonduplicate tweets. The most common terms found within the reduced pain tween corpus included “feel” (n=1504), “don’t” (n=702), “love” (n=649), “can’t” (n=543), “ass” (n=374), “time” (n=340), “life” (n=328), “lol” (n=327), “hurt” (n=294), and “people” (n=288) (Multimedia Appendix 2). There were a total of 14,877 terms that were contained within the reduced pain tween corpus and these terms were connected across 451,209 edges.

**Figure 1:**
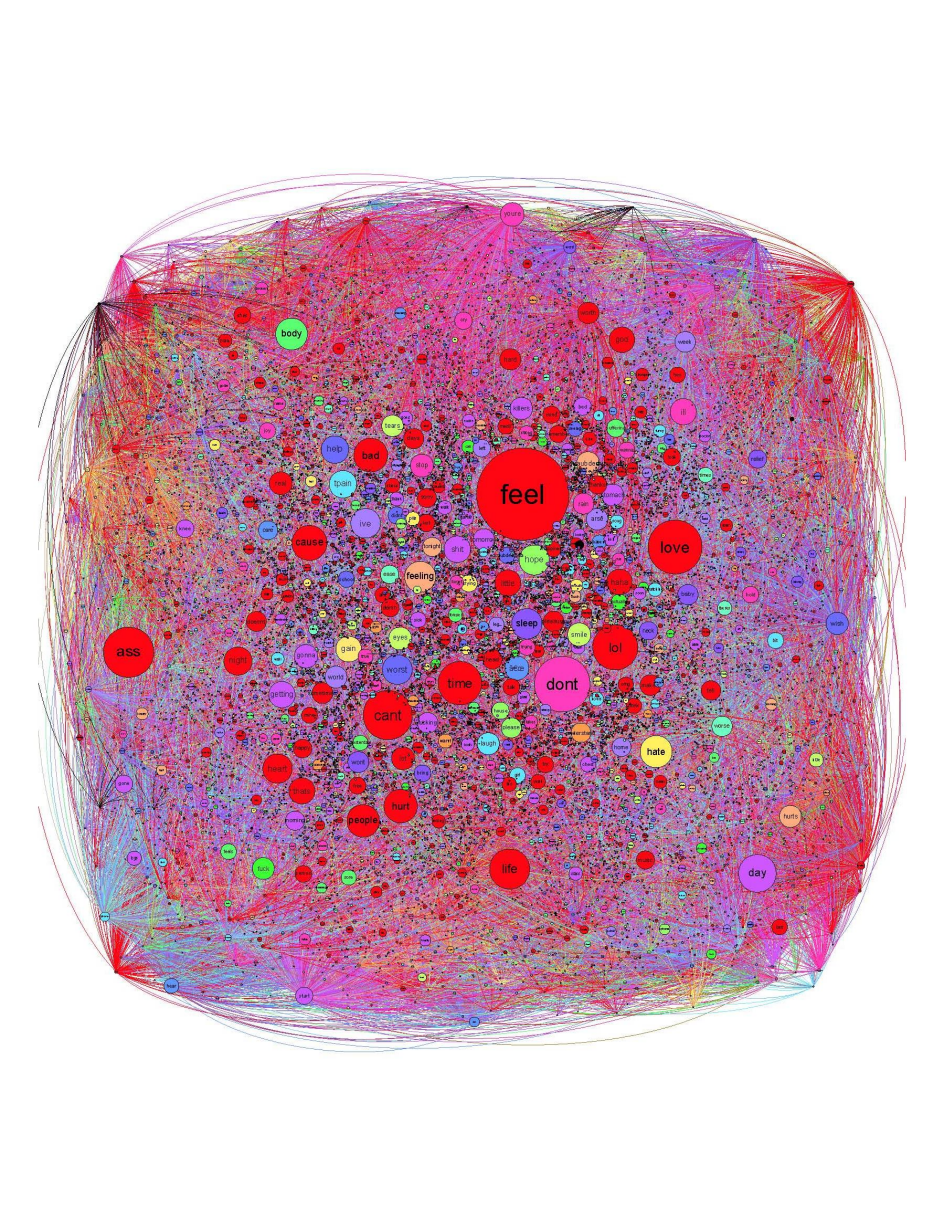
Graph of reduced pain-related tweet corpus. Each term contained within corpus is represented by a point; point size corresponds to the total degree centrality of the associated term. The color of each point indicates membership to a modularity community. Whenever a term is associated with another term within a given tweet, the 2 points are connected by a line, or edge; edge width corresponds to the frequency of association between the 2 connected terms.

**Figure 2:**
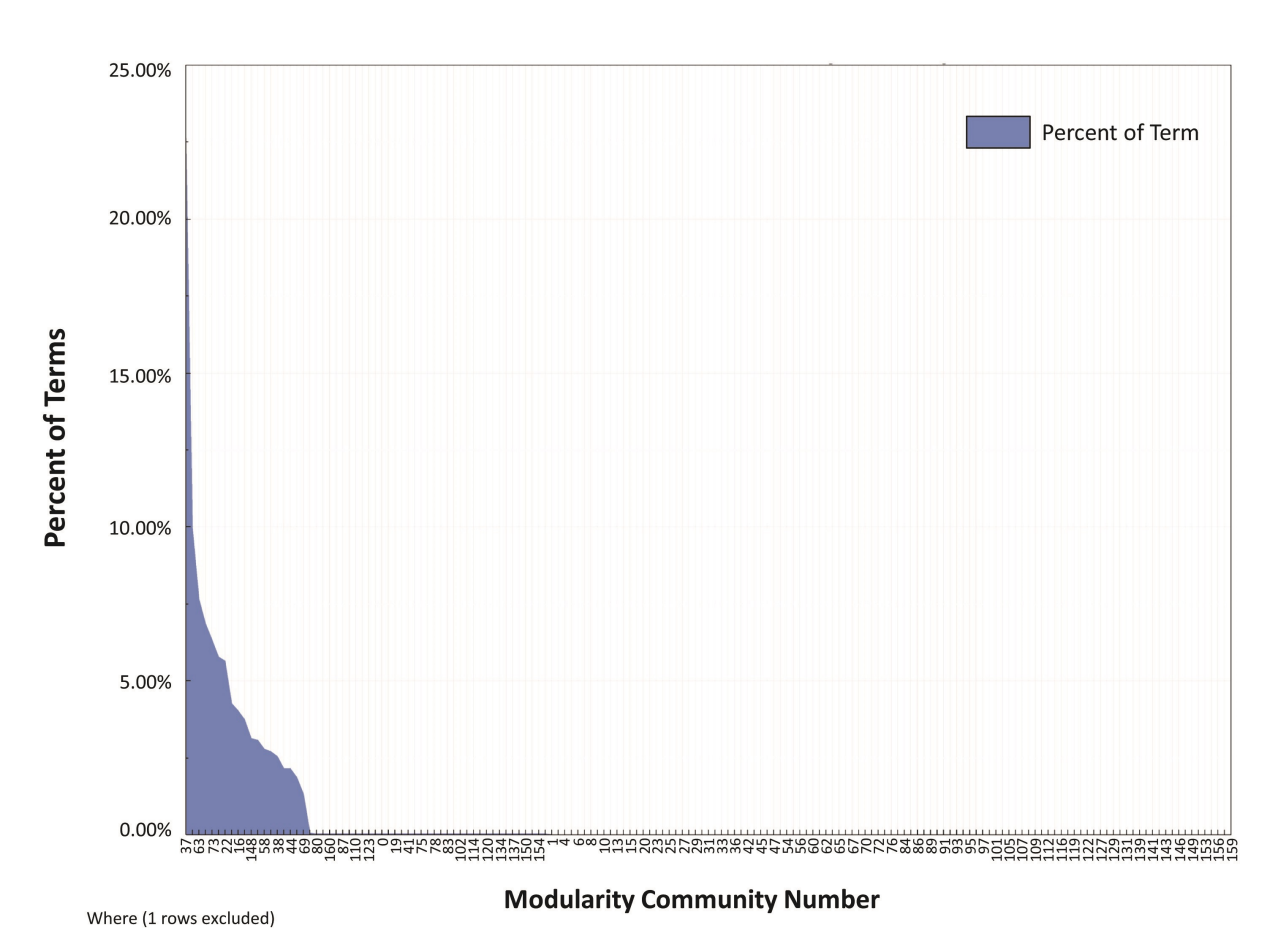
Percentage of terms contained within 161 modularity communities.

The average degree centrality of the reduced pain tween corpus graph was 60.7, with total degree centrality counts for individual terms ranging from 0 to 5652 with a median of 18 (Figure 1). Terms with the highest total degree centrality included “feel” (degree centrality=5652), “don’t” (degree centrality=3375), “love” (degree centrality=3274), “ass” (degree centrality=3049), and “can’t” (degree centrality=2983) (Multimedia Appendix 3). The most common associations between terms, as a function of edge weights, included “laugh” and “watching” (edge weight=566), “don’t” and “feel” (edge weight=395), and “uploaded” and “video” (edge weight=361) (Table 1). A total of 161 modulus-based communities were detected using Louvain’s algorithm (Figure 2). The 10 most common modulus communities accounted for 77% of all terms.

**Table 1:** Edge weights of frequent associations between terms.

Rank	Term 1	Term 2	Edge weight
1	Laugh	Watching	566
2	Don’t	Feel	395
3	Uploaded	Video	361
4	(Name)	Laugh	335
5	Hart	Laugh	310
6	Feel	Lol	283
7	Feel	Love	276
8	Cant	Feel	222
9	Hart	Kevin	200
10	“ ”	Feel	183
11	Waking	Worst	171
12	Baby	Bring	166
13	Hope	Running	164
14	House	Running	163
15	Please	Running	161
16	Chicago	Running	160
17	Marathon	Running	160
18	Miles	Running	160
19	iPhone	Temper	158
20	Carriers	iPhone	158
21	Hope	iPhone	158
22	iPhone	Margin	158
23	(Name)	Watching	155
24	Carriers	Temper	147
25	Hope	Temper	147

#### Sentiment Analysis

The sentiment classifier was validated in stages. In the first stage, the rule-based classifier, which was originally intended for classifying a broad array of text topics, was tested on 3 test sets: a 1500-tweet corpus based on a search for “happy,” a 1500-tweet corpus based on a search for “sad,” and a 1500-tweet corpus based on a search for “apple.” The rule-based classifier identified 92.67% (1390/1500 of “happy” tweets as positive in sentiment, 19.53% (293/1500) of “sad” tweets as positive in sentiment, and 38.32% (575/1500) of “apple” tweets as positive in sentiment. The naïve Bayesian classifier, which was specifically trained on tweets pertaining to pain, identified 89.64% (1345/1500) of the “happy” tweets as positive in sentiment, 69.7% (1046/1500) of “sad” tweets as positive in sentiment, and 90.24% (1354/1500) of “apple” tweets as positive in sentiment (see Multimedia Appendix 4).

In the second validation stage, the sentiment classifier was tested on a holdout set of 100 tweets from the pain tween corpus not previously used for training of the naïve Bayesian component. When rated by humans, this test set contained 38% (38/100) (author PJT), 37% (37/100) (author RG), and 19% (19/100) (author MG) positive tweets depending on the rater, with a Cohen’s kappa of .42, suggesting low to moderate interrater agreement. The rule-based component identified 42% (42/100) of these tweets as positive and the naïve Bayesian component identified 38% (38/100) as positive, with a Cohen’s kappa between the 2 components of .16. When combined with the naïve Bayesian component to create the final hybrid classifier, a total of 39% (39/100) of the pain tween corpus test-set tweets were rated as positive in sentiment. The Cohen’s kappa for the hybrid, rule-based, and naïve Bayesian classifier was .382, and for the human raters and the hybrid classifier was .317 (Multimedia Appendix 3).

**Figure 3:**
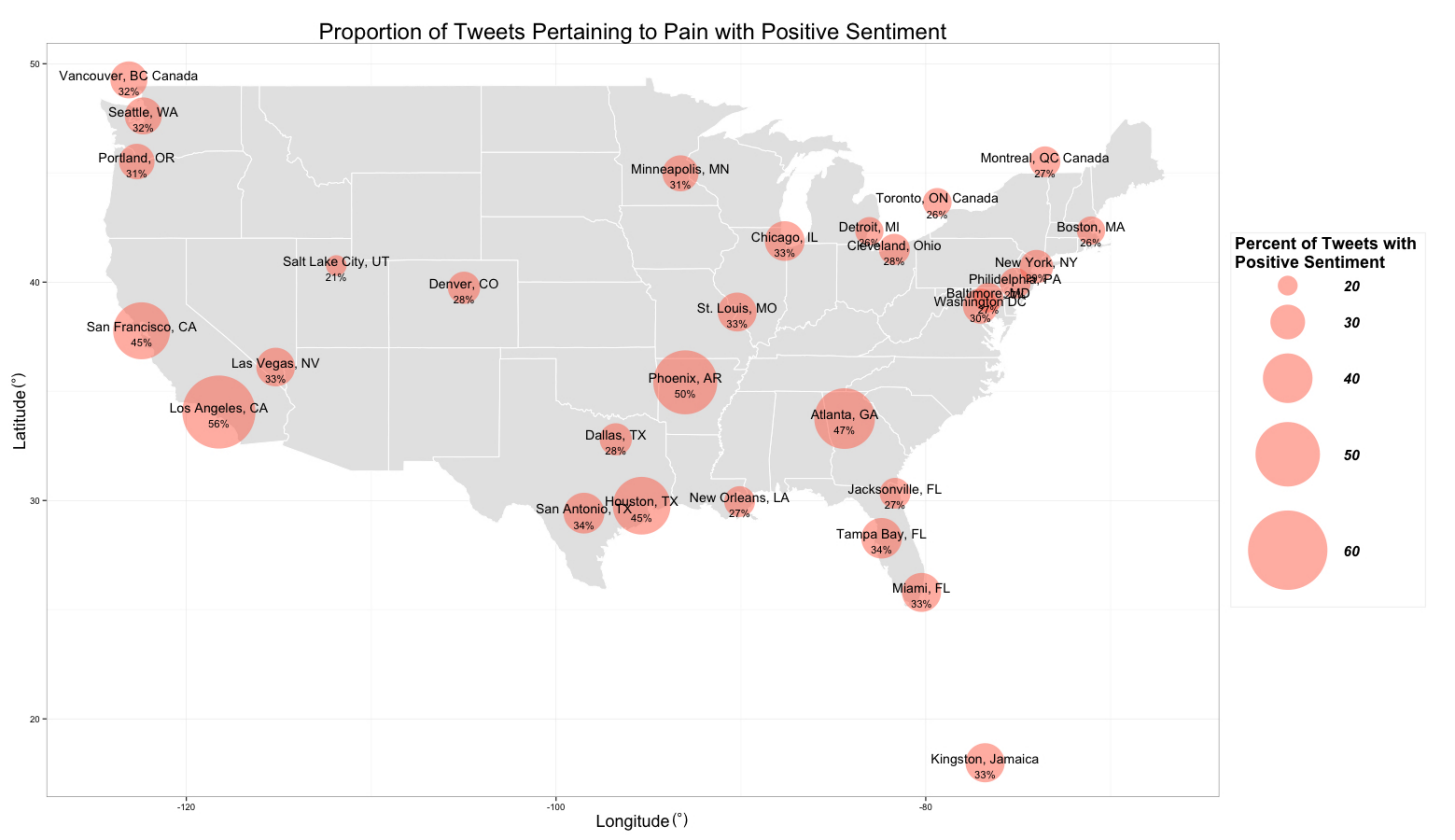
Percentage of pain-related tweets with positive sentiment in selected North American cities. Larger diameter circles indicate higher proportions of positive sentiment in tweets containing the term “pain.”.

Sentiment analysis was conducted on the entire pain tween corpus of 65,410 tweets. Sentiment scores of pain-related tweets were first compared among cities. The proportion of tweets with positive sentiment ranged from 13.13% (197/1500) in Manila, Philippines, to 55.73% (836/1500) in Los Angeles, California, with a median of 29% (Figure 3). There was a statistically significant difference in the proportion of pain-related tweets with positive sentiment among the 49 tested cities (*P*<.001).

**Figure 4:**
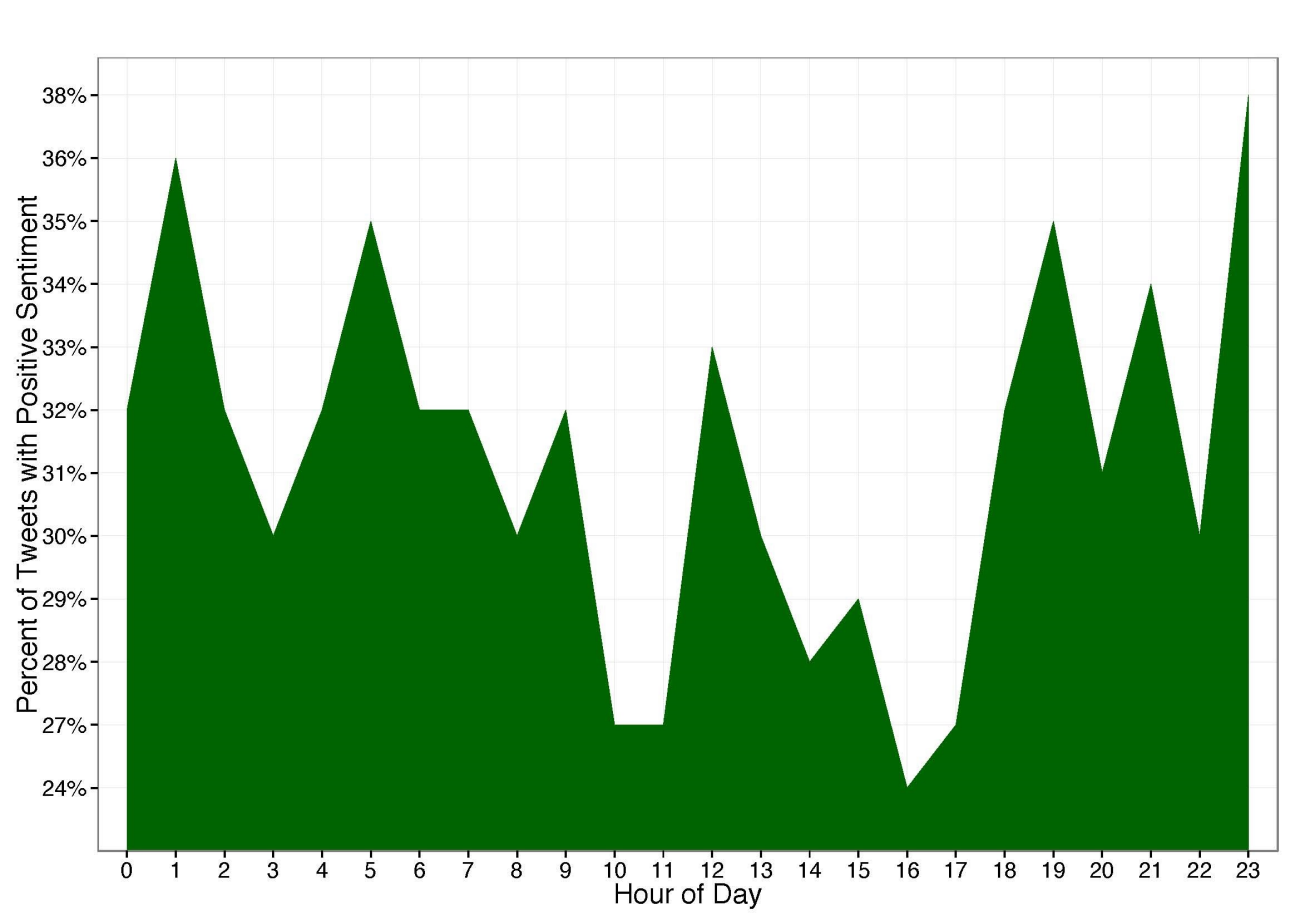
Percentage of pain-related tweets that contained date and time stamps with positive sentiment over a 24-hour period. Times were adjusted from UTC to local times according to geographic location.

Sentiment scores of pain-related tweets were compared across a 24-hour period (Multimedia Appendix 5). The proportion of tweets with positive sentiment ranged from 23.88% (833/3488) at 1600 to 38.25% (469/1226) at 2400, with a median of 32% (Figure 4). There was a statistically significant difference in the proportion of pain-related tweets with positive sentiment across the 24-hour period (*P*<.001).

Correlations between city-level demographic and climate characteristics and the percentage of pain-related tweets with positive sentiment were examined as an exploratory analysis (Table 2). Statistically significant correlations were observed between the percentage of positive tweets and the percentage of individuals without health insurance (ρ=.476, *P=*.02), average high temperature for September (ρ=.425, *P*=.03), and the latitude of the city (ρ=–.42, *P=*.04).

**Table 2:** Spearman rank correlations (ρ) between proportion of positive tweets and city-level demographic and climate data.

Variable	ρ	*P*
Percentage without health insurance	.476	.02
Average high temp in September	.425	.03
Latitude	–.420	.04
Longitude	–.358	.08
Average precipitation days in September	–.305	.14
% High school graduate	–.198	.35
% Bachelor’s degree or higher	.180	.40
Individuals below poverty level	–.169	.42
Median age	.166	.43
Population density	–.111	.60
Median household income	.108	.61
Population	–.018	.93

### Community Structure of Twitter-Based Social Networks Related to Pain

**Figure 5:**
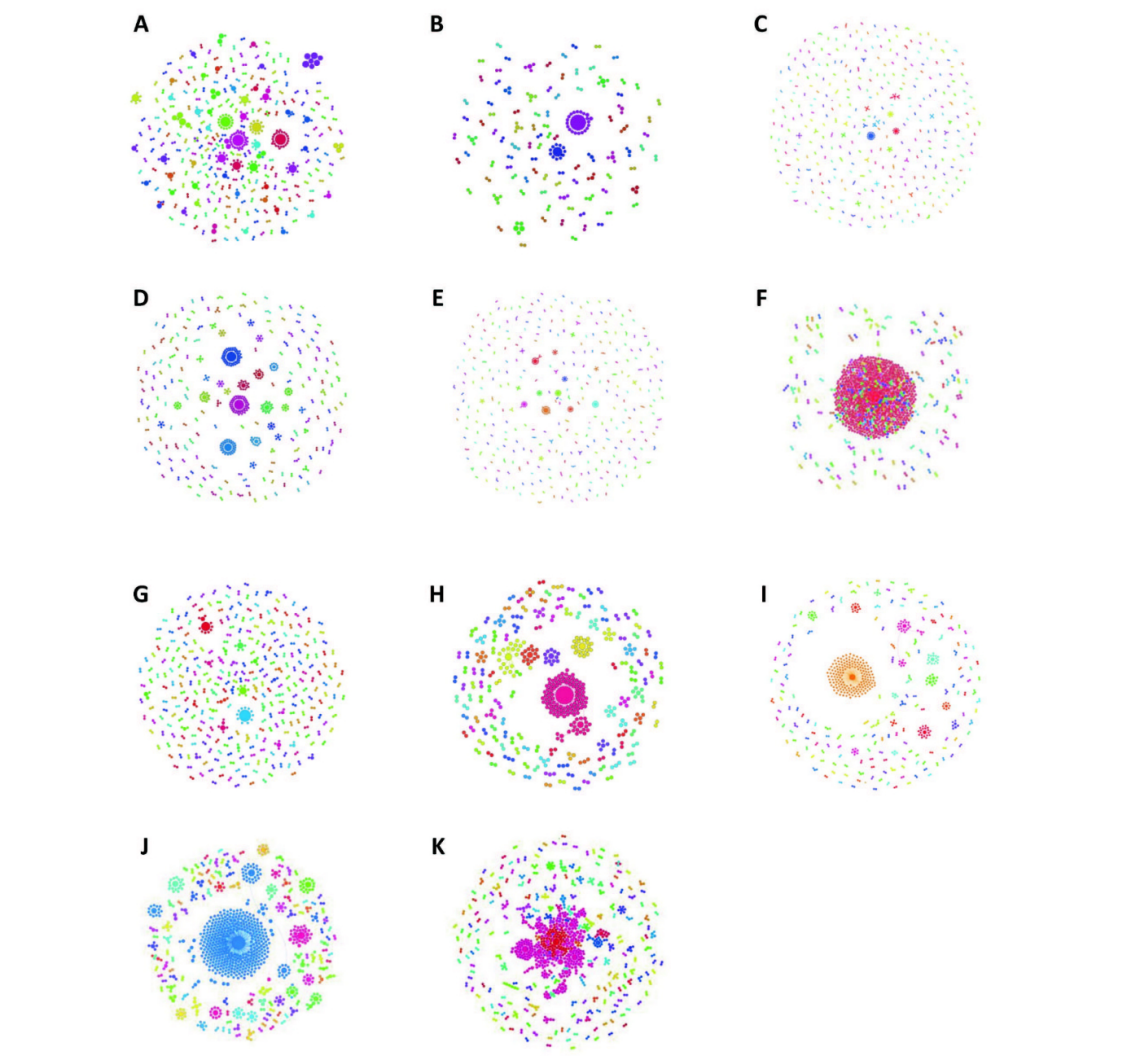
Panel of retweet networks for (A) pain, (B) #pain, (C) happy, (D) excitement, (E) sad, (F) fear, (G) tired, (H) anguish, (I) apple, (J) Manchester United, and (K) Obama. Each circle indicates a node, or Twitter user, and each line connecting the circles represents an edge, or a mention of 1 user in the tweet of another. Each edge is directional in that it “points” from the originating Twitter user to the recipient Twitter user. Node size reflects the degree centrality of the node, line thickness reflects the number of connections between nodes, and color reflects the connectedness community of a node.

**Figure 6:**
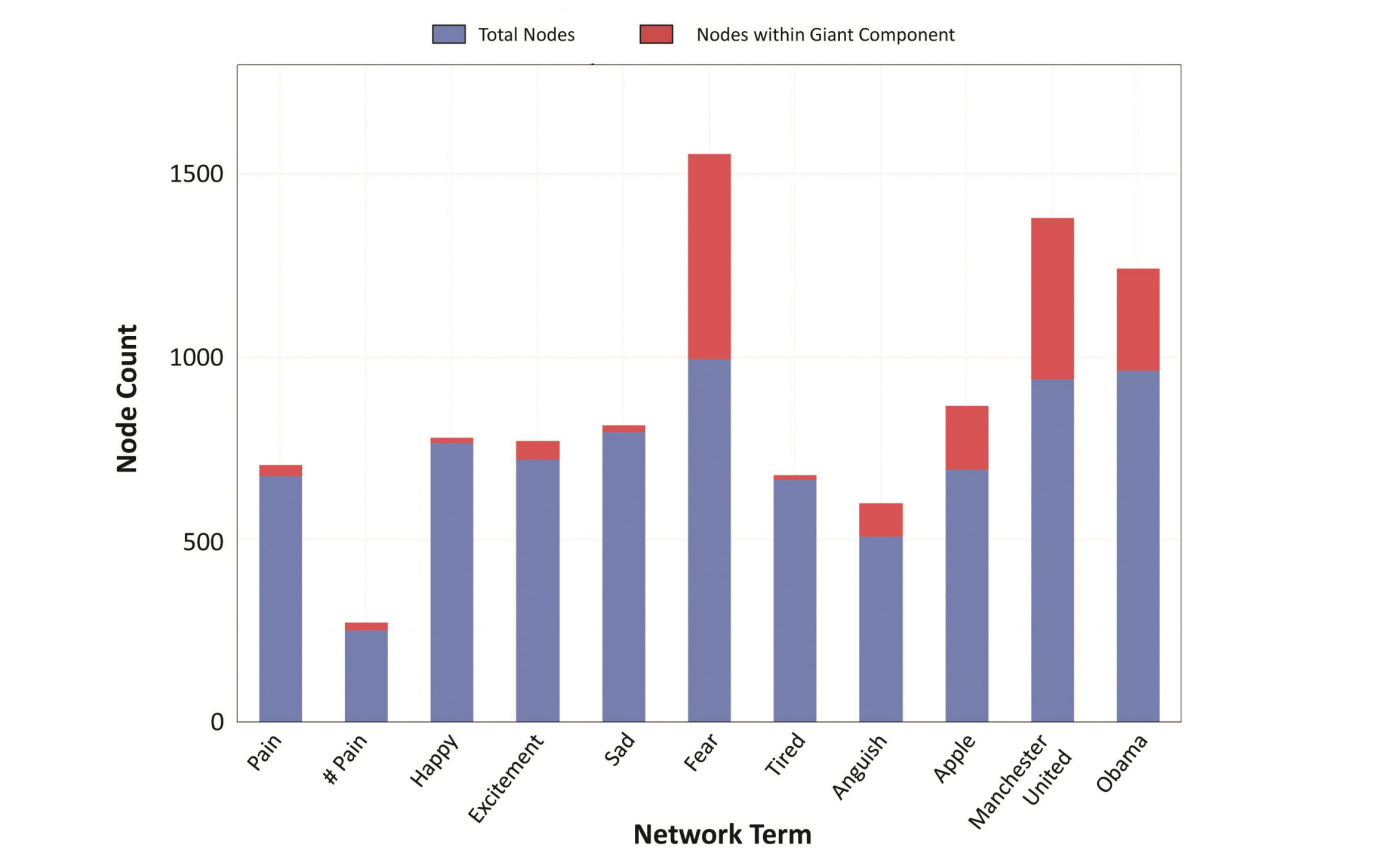
Total (blue) and giant component (red) nodes within retweet networks.

Of 16,500 tweets equally distributed across 11 search terms, 48.28 % (7967/16,500) were involved in retweet networks. By visual analysis of the retweet networks, those pertaining to “pain” exhibited greater sparsity and lower connectedness than did those pertaining to “apple,” “Manchester United,” and “Obama” (Figure 5). The Obama network had the greatest number of retweeting nodes (964), and the Manchester United network had the greatest number of edges (n=827) (Multimedia Appendix 6). Network diameter, average path length, and network density did not differ greatly between the compared networks. The number of weakly connected network components, in proportion to the total number of nodes, was greater for emotional terms when compared with specific objects, ranging from a minimum of 0.14 for Manchester United to between 0.37 for pain, 0.43 for happy, and 0.45 for tired. By contrast, the objective terms overall maintained the greatest percentage of their nodes within the giant component (Figure 6). Manchester United’s network maintained 47% percent of its nodes within the giant component, followed by 29% for Obama and 25% for apple. The emotional terms exhibited lower percentages at 9% for #pain, 4% for pain, 3% for sad, and 2% for happy. An important exception to this trend is the network for fear, which maintained 56% of its nodes within the giant component

**Figure 7:**
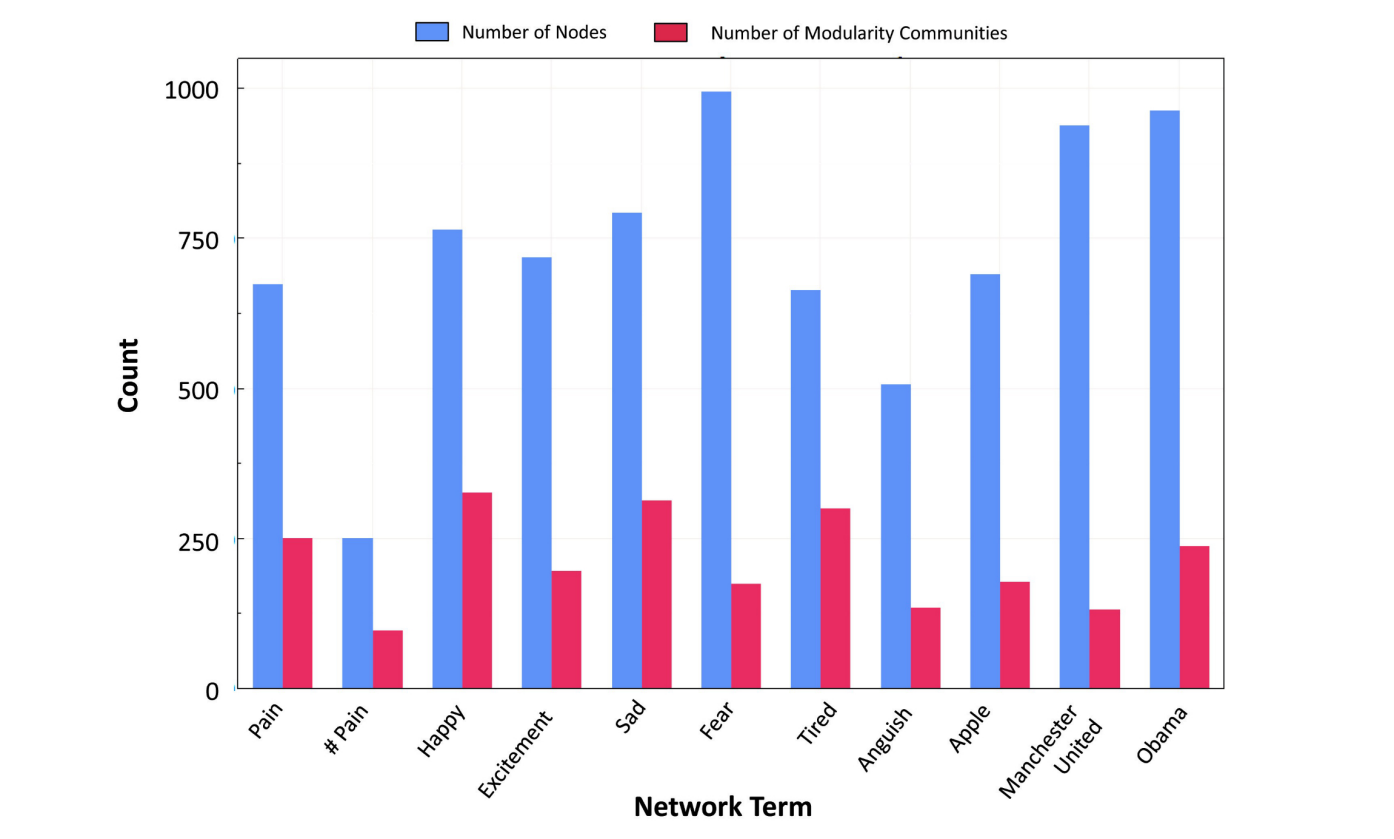
Number of nodes (blue) and modularity communities (red) per retweet network.

Similar to the results for weakly connected network components, the number of modularity communities in proportion to node count was greater for emotional terms such as tired (0.45), happy (0.43), and sad (0.4) when compared with objective terms such as apple (0.26), Obama (0.25), and Manchester United (0.14) (Figure 7). Maximum in-degree centrality scores were greater than out-degree centrality for all terms, although the median numbers for all centrality scores remained between 0 and 1 for all terms (Multimedia Appendix 7). Maximum in-degree centrality scores were greater for objective terms in comparison with emotional terms. In particular, there were statistically significant differences between “apple” and “pain” (mean score difference=−65, *P=*.003, effect size=0.10), “excitement” and “pain” (mean score difference=−70, *P*=.001, effect size=0.10), “Manchester United” and “pain” (mean score difference=−167, *P*<.001, effect size=0.23), and “fear” and “pain” (mean score difference=−175, *P*<.001, effect size=0.23) for in-degree centrality. For out-degree centrality, there were statistically significant differences between “Manchester United” and “pain” (mean score difference=182, *P*<.0001, effect size=0.25), “fear” and “pain” (mean score difference=163, *P*<.001, effect size=0.21), “Obama” and “pain” (mean score difference=79, *P*=<.001 effect size=0.10), and “apple” and “pain” (mean score difference=65, *P*=.002, effect size=0.10). For total degree centrality, there were only statistically significant differences between “Obama” and “pain” (mean score difference=79, *P*<.001, effect size=0.13), and tired and pain (mean score difference=−37, *P=*.002, effect size=0.10) (Multimedia Appendix 8).

In examining the frequency of other emotional and objective terms from the 100 sampled retweeters in the pain term network, we first identified 5967 other tweets published by these individuals. Notably, the term “pain” was mentioned only 35 times within this sample corpus (Table 3). The term “happy” had more than a 2-fold increase in frequency compared with “pain,” and “sad” and “fear” were represented at rates of 86% and 69% of that of pain. Despite their more complicated retweet network structures, the terms for “apple” (3%), “Manchester United” (0%), and “Obama” (14%) were found at substantially lower frequencies in proportion to pain than were the emotional terms.

**Table 3:** Occurrences of emotive terms in 100-user sample of pain network tweeters.^a^

Term	Frequency	Frequency in proportion to pain
Pain	35	1
Happy	73	2.09
Excitement	1	0.03
Sad	30	0.86
Fear	24	0.69
Tired	10	0.29
Anguish	0	0.00
Apple	1	0.03
Manchester	0	0.00
Obama	5	0.14

^a^ Sampled 100 users from pain term network who submitted a tweet containing “pain” and a retweet or mention to an individual. Requested up to 100 of the most recent tweets from each of these individuals. 5967 tweets collected. Searched all text for these terms.

## Discussion

The results presented here suggest that pain-related tweets carry special characteristics reflecting unique content and their communication among tweeters. The majority of tweets appear to describe pain within the context of relationships, although there were certainly a number of themes denoting specific physical pain. These data support the hypothesis that discussions of pain on Twitter do indeed focus on a range of both physical and nonphysical topics and not simply as a medical condition. Approximately one-third of pain-related tweets were quantified as containing a positive overall sentiment, a proportion that differed by geographic location and the hour of the day and supports our second hypothesis of a mixture of positive and negative sentiment in pain-related tweets. Our results also support the hypothesis of a unique connectivity pattern of retweet networks pertaining to pain.

Automated content analysis of pain-related tweets offers several potential applications to researchers, policymakers, and health care professionals. For instance, potential associations between biopsychosocial factors and tweet content may assist in the prediction of acute and chronic pain outcomes. More in-depth explorations of tweets related to pain may better differentiate physical versus emotional sources of pain through the use of extremely large datasets of tweets, although such differentiations would require calibration via external methods of data collection to ascribe the content to emotional versus physical sources with any degree of certainty. The volume of tweets available, coupled with their time and location tags, may permit analyses of seasonal and temporal shifts in pain density and their association with environmental and geopolitical events [22]. Indeed, prior work suggests that Twitter sentiment scores may lead public opinion surveys by a few days, thus offering an inexpensive way to indirectly assess public perception [68].

It may also be possible to use this method as an epidemiologic platform for ascertaining community health and a barometer of health care needs pertaining to pain in a manner similar to the experimental use of Twitter content as an influenza surveillance tool [69]. Such policy-based approaches to pain surveillance could help direct the allocation of pain management resources in time and location. Supplementing conduct of surveys of unmet pain needs on an annual or semiannual basis, the methods presented here could permit monthly, or even weekly, reviews of the effects of pain policy changes. Although limited by several critical factors, such as differentiation between acute and chronic pain, collection of data from a skewed population of social media users, and contextual relationships pertaining to pain, a streaming measure of the use and sentiment of emotive terms such as pain nevertheless may offer a low-cost, real-time supplement to such methods. Although such an initiative may only offer association-level data that may be of purely academic interest, it is at least feasible that calibration of such methods against established, robust (and sometimes cost-prohibitive) data collection measures may attach some value to such a social media-based approach to data collection, especially within targeted populations such as teenagers and Generation Y members. As suggested by Greaves et al [70], Twitter-based sentiment analyses could also help detect poor quality of pain-related health care delivery. This could lead to an efficient, widely deployable adjunct to the current Hospital Consumer Assessment of Healthcare Providers and Systems (HCAHPS) method of assessing patient satisfaction with pain control. Notably, existing work in this particular area points to widespread geographic disparities in patient experience with pain management during hospitalization, as well as agreement between social media ratings of hospitals and HCAHPS measures, thus pointing to an opportunity for social media sampling to supplement existing data collection methods [50,71].

Quantification of sentiment, especially as measured in a 140-character document rife with abbreviations and slang, poses valid questions concerning the accuracy and repeatability of the classifier. Prior work using related methods of sentiment quantification of tweets suggests that such sentiment analysis tracks well with important sociocultural events, although the magnitude of change in sentiment may be small and biased toward increases in negative rather than positive sentiment [16,29]. It is reassuring that such lexical accuracy has been demonstrated using word lists even without the inclusion of a machine learning component and has been reported for sentiment analysis projects targeting emotional constructs such as “happiness” [72].

Our analyses on correlations between the proportion of positive tweets and city-level demographics were intended to be exploratory given the lack of rigor in the selection of sampled cities. The correlations between average high temperature and latitude are reasonable given that higher temperatures and lower latitudes may be associated with greater sunlight exposure and more positive affect [58]. The presence of geographic differences in tweet sentiment is in keeping with very recent work comparing the “happiness” of cities in the United States by measuring the overall sentiment of 10 million geotagged tweets collected in 2011, although such comparisons are limited to the presence of differences given that this study did not examine the role of latitudes and temperatures in association with city ”happiness” [20]. When reviewing such matters, it is important to consider the risk of ecological fallacy given the simultaneous measures of individuals and groups.

Our results suggest that the sentiment of tweets pertaining to pain differs over the 24-hour interval. This is in keeping with prior work by Thelwall et al [29], which suggests that sentiment seems most negative in the late morning and late afternoons. However, this contrasts with work by Dodd et al [18], which showed small increases in positive sentiment between the hours of 0500 and 0600 and again between 1900 and 2000. Our results similarly show spikes in positive sentiment at 0500 and 2000, as well as supplemental spikes at 1200 and sustained increases after 2000. Interestingly, in the United States, the time intervals with lowest sentiment for pain-related tweets coincide with the hours that frequently precede mealtime.

One interesting observation noted in this exploratory analysis was the association between positive sentiment of pain-related tweets and areas with a high percentage of individuals without health insurance. It is possible that the skewed demographics of social media users may also be those without health insurance, which would be incongruent with prior data associating chronic pain with access to health services [73]. This may further point to the discrepancy of pain as a disease state versus the representation of the concept of pain within social media platforms.

Aside from content analysis, examination of pain-related tweets can also uncover information about the online social networks of those tweeters who tweet about pain as a matter of discourse. In recent years, multiple teams have explored social media platforms as they relate to social support systems for physical and mental health challenges [12,23,26,42-46]. Indeed, an automated social network analysis of pain-related tweets of patients may serve to quantify and monitor treatment progress for many potential patient-centered outcomes. Such approaches can be simultaneously applied to individual patients and entire communities, thus helping policymakers gauge the effectiveness of large-scale treatment interventions as well as provide decision support regarding resource allocation.

Retweet patterns for tweets pertaining to “pain” yield smaller discussion communities than do tweets on objective subjects. Those users involved in pain-related discussions were weakly connected through giant components of smaller size and were more likely to participate instead in one of a larger number of smaller modularity communities. Taken together, these results suggest that tweeters tend not to promote statements from others pertaining to pain, as they might with tweets on subjects such as sports or politics. Notably, this is not terribly dissimilar in structure to retweet networks for ”apple” and even ”Manchester United,” although the component sizes for pain retweets are indeed much smaller.

In contrast to prior work on the use of social media outlets for social support systems, our results suggest that such publications pertaining to pain may not trigger social media equivalents of “conversations” as they might if one were making a statement concerning sports or politics [12,23,26,41-46]. For pain and other emotional terms, most retweets were “dead end” expressions with a path length of 1. However, for objective term networks, retweets seemed to “echo” prior content across multiple successive communities, leading to longer path lengths. Tweeters expecting responses to tweets about pain may thus be surprised at a perceived lack of empathy from the Twitterverse.

Saito and Masuda [74] have demonstrated 2 types of popular tweeters: the first has many followers but follows only a small number of individuals themselves, whereas the second maintains large communities of followers and followed sources. The pain and #pain retweet networks mostly followed the type 1 schematic of Saito and Masuda, and the type 2 schematic predominated for the objective term networks. Others have differentiated retweet behaviors into “broadcasters,” or those with many followers but who follow few sources themselves, and “miscreants,” or those with few followers but who follow many sources. Here again, we see that pain-related tweets follow a miscreant pattern of retweets, whereas objective term networks, which echo earlier tweets into multiple communities, follow the broadcasters pattern of retweeting [9].

The lack of retweeting about pain may indeed limit the utility of Twitter, at least as a limited dataset, in studying pain-related discussions. On the other hand, the presence of tweets about pain in the context of an overall low prevalence on the topic may offer an important insight into a given tweeters’ focus on pain. To this end, earlier work on social media and chronic medical conditions suggests that alternative social network media, such as Facebook, contains more health care groups than can be found on Twitter [75]. Prior work with tweets pertaining to incontinence have noted a lack of “useful content,” suggesting that some medically oriented Twitter content lacks a suitable substrate for conversation [21]. Given the findings by Kumar et al [76] suggesting that topics of discussion heavily influence a user’s interest in participating in Twitter-based discussions, it may simply be that tweeters are uninterested in discussing topics related to pain. Regardless, the observed lack of social promotion of pain-related tweets may limit the utility of Twitter as a medium for promoting social interactions in those with impaired social networking due to chronic pain.

This work opens several interesting possibilities pertaining to pain research. The volume of tweets available, coupled with their time and location tags, may permit analyses of seasonal and temporal shifts in pain density and their association with environmental and geopolitical events [57]. Such policy-based approaches to pain surveillance could help direct allocation of pain management resources in time and location in a manner similar to that of Twitter-based resource allocation during natural disasters [4,16,17,23-28]. Instead of conducting surveys of unmet pain needs on an annual or semiannual basis, the methods presented here could permit monthly, or even weekly, reviews of the effects of pain policy changes. As suggested by Greaves et al [70], Twitter-based sentiment analyses could also help detect poor quality of pain-related health care delivery. However, such benefits must be viewed in the context of the complexity of the task of searching through large volumes of tweets to identify pain-related material and then processing this material into relevant information that can be used for research and/or decision support.

Given the scope of our project, we accumulated several limitations pertaining to methods available for Twitter-based research. First, given that Twitter is predominantly used by younger individuals more comfortable with technology, our results do not account for large swaths of the general population. A 2012 survey by the Pew Research Center suggests that 16% of Internet users use Twitter and that Twitter “is especially appealing to” adults aged 18 to 29 years, African-Americans, and urban residents [77]. However, this is also true for volunteer studies in pain research, which traditionally sample primarily from young adult populations [73]. Our study examined only public tweets; it is certainly possible that the preceding results may be skewed even among social media users given that social media users who tweet about their pain experiences may choose to keep their postings private due to the personal nature of this topic. Our work was similarly limited in its use of only English-language tweets; therefore, our data are likely not representative of all tweets originating from cities that are primarily non-English speaking. In examining groups of terms, our use of default community modularity coefficients may have led to inappropriately large populations of terms in the upper-tier communities. However, this approach also permitted encapsulation of broader topics, and minimized the chance of having topics populate multiple communities. The interrater kappa coefficients were admittedly low, but are in keeping with prior interrater annotation scores for sentiment analysis [11,56,57] that points to the subjective nature of sentiment analysis. The decision to study the selected cities was made empirically and was based on an effort to examine cities across a range of geographic regions and rural versus urban characteristics. Although a larger sample with a broader range of characteristics would have been attractive, this was unrealistic given that the size of the studied pain tween corpus grossly strained computing resources.

In conclusion, our results suggest that graph and sentiment analysis of pain-related tweets can offer important insights into the roles of pain throughout the social media discourse prevalent in today’s society. Indeed, the preponderance of emotional and psychological pain references identified by our study suggests that future studies focusing on terms related to the physical manifestation of pain are necessary to explore this important aspect of pain research. Furthermore, the actual application of future semantic network analyses should include enhancements such as stemming, n-gramming, and synonym lists to improve the accuracy of their classifications. Further work is necessary to discern how geopolitical events and seasonal changes affect tweeters’ perceptions of pain [37] and how such perceptions affect therapies for pain.

## Multimedia Appendix 1

Pain tweet corpus generation.

## Multimedia Appendix 2

Most common terms in reduced pain tweet corpus.

## Multimedia Appendix 3

Terms with highest total degree centrality.

## Multimedia Appendix 4

Agreement statistics between human rates and classification methods for additional details of classifier performance, including specific information on sensitivity and specificity.

## Multimedia Appendix 5

Pain-related tweet volume by hour of day.

## Multimedia Appendix 6

Graph-level metrics.

## Multimedia Appendix 7

Node-level metrics.

## Multimedia Appendix 8

Effect sizes of in-degree, out-degree, and total degree centralities of retweet networks.

## Abbreviations

API: application programming interface
GPS: Global Positioning System
HCAHPS: Hospital Consumer Assessment of Healthcare Providers and Systems
