# Supplementary material for: The Painful Tweet: Text, Sentiment, and Community Structure Analyses of Tweets Pertaining to Pain
Source: J Med Internet Res. 2015 Apr 2;17(4):e84. doi: 10.2196/jmir.3769 (PMC4400316; doi:10.2196/jmir.3769)
Supplement: Supplementary file 1 [file jmir_v17i4e84_app1.pdf]

Multimedia Appendix 1

Pain Tweet Corpus Generation

Tweets were collected using the TwitteR 1.1 (http://cran.r-project.org/web/packages/twitteR/) and plyR 1.8 [Hadley Wickham 2011. The split-apply-combine strategy for data analysis. Journal of Statistical Software 2012;40(1):1-29. http://www.jstatsoft.org/v40/i01/] packages for R. Notably, the Twitter API searches not for the presence of the four letters p-a-i-n, but rather for the term “pain” as an independent entity. Stemmed versions of the term pain, such as painful, pained, or painting were not searched to avoid ambiguity in the returned results.

The search was conducted on a single day in the last 2 weeks of the month, and as per the Twitter API, collected tweets up to 7 days prior to the search date. The latitude and longitude associated with each tweet reflect the physical location of the tweeters at the time of tweeting rather than the home city or other location of the Twitter account belonging to that individual. Because each tweet was collected on the basis of a city-specific search, each tweet was tagged with the location of that city. Although up to 1500 tweets could be downloaded for each city’s search, limitations imposed by location, time, and English language restriction sometimes limited the number of tweets downloaded for a given city.

All tweets were converted to the UTF-8 text format for uniform processing. The pain tweet corpus was used for sentiment analysis. A reduced version of the pain tweet corpus, whereby all duplicate tweets and retweets were removed, was used for the graph analysis.

Graph Analysis

Graphs are a generalized form of networks whereby only the presence or absence of an edge (A is connected to B) is considered, whereas networks consider the order of connection of edges as relevant (A to B, but not necessarily B to A) [1]. Common graph analysis methods include the calculation of a node's connectedness to other nodes, as well as the identification of subgroups of nodes, or communities*,* within large graphs. Connectedness is commonly measured by degree centrality, which simply sums how many different nodes to which a given node connects. Community identification is analogous to clustering, whereby nodes (analogous to observations) are grouped together with other similar nodes based on the patterns of connections between nodes.

For graph analysis of tweet content, we used a reduced pain tweet corpus whereby all duplicate tweets, including retweets, were removed. This reduction was performed to emphasize the diversity of terms present rather than the magnitude of an effect for a given location or time. Retweet terms using "RT" and "via" prefixes were filtered from each tweet, as were direct messages using the "@" symbol, punctuation, numbers, html links, and a collection of English language stop words, which included the terms "pain" and "amp." All terms were converted to lower case for uniformity of search. Stemming, or the process of removing suffixes of words so as not to differentiate between tenses and uses (eg run versus running), was not employed to avoid ambiguity on implementation of different forms of a given term within the reduced pain tweet corpus. For the same reason, we elected not to include a synonym list. N-grams, whereby terms are grouped into collections of 2, 3, 4, or n sequential terms, were not used due to pragmatic issues of extremely prolonged computational times.

The reduced pain tweet corpus was then converted into a term document matrix using the tm package for R (http://cran.r-project.org/web/packages/tm/index.html). The term document matrix is a matrix whereby the rows represent the tweets themselves (documents), and the terms represent a very wide array of columns with each word contained within the corpus representing a single column [2]. If a term is used within a given tweet, the corresponding tweet-word cell is filled in with the frequency of that term's usage within said tweet.

Following creation of the term-document matrix, we created an adjacency matrix of terms by multiplying the term-document matrix by its transpose, with diagonals set to zero [3]. The term-document matrix was multiplied by a "flipped" version of itself so that instead of the matrix representing counts of terms within tweets, it now represented counts of terms associated with other terms. The adjacency matrix was a term-by-term matrix, where each cell denoted the number of times two terms was found within a given tweet. This matrix forms the basis of the graph analysis, where nodes represent those terms found in row and column headings, and edges represent the frequency with which the nodes, or terms, were associated with each other within a given tweet.

Given the size of the reduced pain tweet corpus, we chose to use a community detection algorithm based on the Louvain method [4]. The Louvain method is one of many different approaches to detecting communities of nodes within a network [5–8]. This particular method is predicated on community identification via modularity, whereby a community's modularity reflects the density of links within a community compared to the density of links between different communities [9,10]. Briefly, the Louvain heuristic first identifies small communities of terms by optimizing the modularity of possible community structures. This method then aggregates these term-based communities, resulting in a

meta-network whose component nodes represent the initial term-based communities themselves, again using modularity optimization. These two steps are iteratively repeated until a maximum modularity is reached.

Given the primary aim of exploring the content of tweets explicitly containing the word "pain," we elected not to include a synonym list in our graph-analytic approach out of concern that this might contaminate the associations among terms. This approach had the benefit of avoiding even the perception of bias inherent in the use of synonym lists. Recognizing the possibility for duplication of concepts due to use of slang and near-equivalent terms for concept description, we elected instead to report lists of the top items for each category of result such that the reader may consider such associations for themselves, and consider the use of synonyms in future work based on the results reported here. Ideally, we would have pursued a variety of pain-related terms in our search to both expand the number of included tweets as well as to compare the content, sentiment, and social networks of tweeters among these terms. Our choice reflected the primary aim of this study, which was to explore how the general term pain was used in tweets. The use of synonyms for pain would have raised further questions about what exactly we were studying. This would have carried very important implications for content analysis as well as sentiment analysis because we would have been unable to untangle the relative contributions from each individual term using the methodologies at hand.

Sentiment Analysis

The Naïve Bayes algorithm is a very simple probabilistic classifier that assumes that the presence or absence of a given word or phrase is completely unrelated to the presence or absence of every other word or phrase [11–15]. This algorithm is considered a supervised machine learning algorithm in that it must be trained on data where the

outcome is known before it can be deployed on data with unknown outcomes. In our implementation, the Naïve Bayes algorithm determined how the presence of each word or phrase, considered independently (or naively), contributes to the probability that the tweet is positive or negative in sentiment. During model creation, training tweets were tokenized as individual terms as well as within sentences. Terms were tagged with a parts-of-speech tagger and aggregated using noun phrase extraction. Predefined stop words were filtered during training. This algorithm was trained using 200 positive tweets and 291 negative tweets sampled from the pain tweet corpus, which were manually scored. The final, hybrid classifier weighted the statistical model 80% and the rule-based model 20% in assigning its final scores.

Collection of Retweet Data

We used the twitteR package to search for retweets. Based on the results of our content analysis, the use of pain and #pain were intended to differentiate between those tweets mentioning pain in routine discourse versus those tweets with pain as a central subject of the tweet as indicated through the use of the hashtag via #pain. Note that this collection of tweets was separate from the pain tweet corpus and reduced pain tweet corpus used for the above content analyses because the number of tweets contained within each search needed to contain equal numbers of tweets across a broad array of topics.

For the collection of tweets returned from each search, a list of those tweets identified as retweets was created by searching for "RT @ " or "via @" followed by the retweeted message using regular expressions calculated using the grep and stringR (version 0.6.2) packages (http://cran.r-project.org/web/packages/stringr/index.html). For each list of retweets, the usernames of the retweeted user and the retweeting user were extracted on a per-tweet basis, such that the posting screen name served as the source node and the

username included in the retweet itself, such as with @reply or @mention, was the recipient node. This listing of source and destination nodes thus formed a list of nodes that were connected with other nodes. The connections between nodes are known as edges in network parlance, and so this listing is known as an edge list. In the event of multiple pairings of source-destination nodes, the frequency of pairings was summed so that the number of source-destination pairings reflected the edge weight. The edge list was converted to a graphml file for export into Gephi 0.8.2 (Gephi Consortium, Paris, France) using the igraph 0.6.5 package [16].

Social Network Analysis Metrics

Node count refers to how many separate people, or nodes, there are, and edge count refers to how many connections between nodes existed within the network. The network diameter is the longest distance, measured in edge count, between any two nodes within the network, and the average path length is the average distance between all pairs of nodes within the network. Network density is a measure of how close the network is to a "complete" network in which every node is connected to every other node and the density is 1.0.

Connected components are subgraphs within a network in which all components are connected to each other [17]. Connected components are considered strongly connected if we must consider the directionality of the connecting edges, and are weakly connected if we are able to ignore the directionality of said edges. The giant component, or the largest of the connected components, within each network was identified, and the number of nodes and edges within each giant component was summed [18].

Modularity communities represent communities of tweeters whereby each tweeter has more communications within their group than with tweeters outside of their group [19,5–7]. The term “modularity” refers to the mathematical algorithm used to canvas the network to identify such communities. This calculation is similar to the communities of associated terms used in the content analysis experiments, although nodes here represent individual twitter users instead of words. Degree centrality scores reflect the number of different nodes to which each node is connected. In-degree and out-degree centrality are variants of degree centrality, and count how many different nodes point to (in-degree) or away from (out-degree) a given node. Centrality metrics were reported as median and range.

Statistical Analyses

iGraph analyses, including degree centrality and modularity community calculations, were conducted using Gephi 0.8.2 (Gephi Consortium). All other statistical analyses were conducted using R 2.15.2. Interrater agreement between test subjects was implemented using Conger’s exact Kappa statistic via the irr package (Gamer A, Lemon J, Fellows I, Singh P. Various coefficients of interrater reliability and agreement. CRAN. July 16, 2012. http://www.r-project.org) for R [20,21]. Accuracy of the classifiers was tested by creating a 2 × 2 table of positive versus negative by positive versus negative sentiment for each classification method versus human reviewer, and then calculating the sensitivity, specificity, precision, accuracy, and F-measure for each table. Tests of the distribution of positive sentiment tweets by city and hour employed the two-sided multiple sample test of equal proportions. City-level correlations were performed using Spearman’s rho. Total degree centrality, in-degree centrality, and out-degree centrality were compared across retweet communities using the Kruskal-Wallis test, with nonparametric multiple

comparisons conducted against the control term of "pain" using the Steel Method [22] with JMP 10.0.02 (SAS Institute, Cary, NC). Effect sizes were reported by dividing the Z-score of the Kruskal-Wallis test by the square root of the sum of the group sizes for each pair of terms tested in the Steel Method. With this effect size metric, 0.1 represents a small effect size, 0.3 a moderate effect size, and 0.5 a large effect size. Given the number of comparisons tested in this study, level of significance was set at $P = .01$.

References

1. Twitter geolocation and its limitations. Available at http://dfreelon.org/2013/05/12/twitter-geolocation-and-its-limitations/. Accessed September 13, 2013. (Archived by WebCite® at http://www.webcitation.org/6PlToQzfw)

2. Manning CD, Raghavan P, Schütze H. Introduction to Information Retrieval. New York, Cambridge University Press; 2008. 978-0521865715

3. Antonellis I, Gallopoulos E. Exploring term-document matrices from matrix models in text mining. Technical Report 2006 arXiv:cs/0602076v1.

4. Blondel VD, Guillaume J-L, Lambiotte R, Lefebvre E. Fast unfolding of communities in large networks. Technical Report 2008 arXiv0803.0476v2.

5. Fortunato S. Community detection in graphs. Physics Rep 2010;486:75-174. 10.1016/j.physrep.2009.11.002

6. Lancichinetti A, Fortunato S. Community detection algorithms: a comparative analysis. Phys Rev 2009 E 80:056117. PMID: 20365053

7. Porter MA, Onnela J-P, Mucha PJ. Communities in Networks. Notices Amer Math Soc 2009;56(9):1082-1097, 1164-1166. 0902.3788

8. Reichardt J, Bornholdt S. Statistical mechanics of community detection. Phys Rev E 2006 74:016110. PMID: 16907154

9. Girvan M, Newman MEJ. Community structure in social and biological networks. Proc Natl Acad Sci USA 2002;99:7821-7826. PMID: 12060727

10. Newman M. Analysis of weighted networks. Phys Rev E 2004 70:056131. PMID: 0407503

11. Al-Aidaroos KM, Bakar AA, Othman Z. Naïve Bayes variants in classification learning. 2010 International Conference on Information Retrieval and Knowledge Management; 2010 Mar 17-18; Shah Alam, Selangor. IEEE; 2010. 10.1109/INFRKM.2010.5466902

12. Elkan C. Naive Bayesian Learning. Department of Computer Science, Harvard University. Adapted from Technical Report No. CS97-557, Department of Computer Science and Engineering, University of California, San Diego, September 1997.

13. Friedman N, Geiger D, Goldszmidt M. Bayesian network classifiers. Machine Learning 1997;29:131-163. 10.1023/A:1007465528199

14. Lahlou FZ, Mountassir A, Benbrahim H, Kassou I. A text classification-based method for context extraction from online review. Eighth International Conference on Intelligent Systems: Theories and Applications; 2013; pp. 1-5. 10.1109/SITA.2013.6560804

15. Sebastiani F. Machine learning in automated text categorization. ACM Comput Surv 2002;34:1-47. 10.1145/505282.505283

16. Csardi G, Nepusz T. The igraph software package for complex network research. InterJournal Complex Systems 2006; p. 1695. citeulike: 3443126

17. Freeman LC. Centrality in social networks conceptual clarification. Social Networks 1978;1:215-239. 10.1016/0378-8733(78)90021-7

18. Molloy M, Reed B. The size of the giant component of a random graph with a given degree sequence. Combinatorics Probability Computing 1998;7:295-305. 10.1017/S0963548398003526

19. Barber MJ. Modularity and community detection in bipartite networks. Phys Rev E 2007 76:066102. PMID: 18233893

20. Conger AJ. Integration and generalization of kappas for multiple raters. Psychol Bull 1980;88:322. 10.1037/0033-2909.88.2.322

21. Fleiss JL. Measuring nominal scale agreement among many raters. Psychol Bull 1971;76:378. 10.1037/h0031619

22. Douglas CE, Michael FA. On distribution-free multiple comparisons in the one-way analysis of variance. Communications Stats Theory Methods 1991;20:127-139. 10.1080/03610929108830487
