# Supplementary material for: The Painful Tweet: Text, Sentiment, and Community Structure Analyses of Tweets Pertaining to Pain
Source: J Med Internet Res. 2015 Apr 2;17(4):e84. doi: 10.2196/jmir.3769 (PMC4400316; doi:10.2196/jmir.3769)
Supplement: Supplementary file 2 [file jmir_v17i4e84_app2.pdf]

Multimedia Appendix 2. Most Common terms in Reduced Pain Tweet Corpus		
**Rank**	**Term**	**Frequency**
1	Feel	1504
2	don’t	702
3	love	649
4	cant	543
5	ass	374
6	time	340
7	life	328
8	lol	327
9	hurt	294
10	people	288
11	â€œ	278
12	cause	271
13	day	267
14	smile	267
15	worst	265
16	bad	252
17	body	245
18	feeling	244
19	heart	243
20	makes	240
21	ive	235
22	hate	218
23	hope	218
24	sleep	213
25	youre	211
