# Supplementary material for: The Painful Tweet: Text, Sentiment, and Community Structure Analyses of Tweets Pertaining to Pain
Source: J Med Internet Res. 2015 Apr 2;17(4):e84. doi: 10.2196/jmir.3769 (PMC4400316; doi:10.2196/jmir.3769)
Supplement: Supplementary file 3 [file jmir_v17i4e84_app3.pdf]

Multimedia Appendix 3. Terms with Highest Total Degree Centrality		
**Rank**	**Term**	**Degree**
1	feel	5652
2	dont	3375
3	love	3274
4	ass	3049
5	cant	2983
6	lol	2733
7	time	2631
8	life	2419
9	day	2264
10	cause	2105
11	bad	2030
12	hurt	2008
13	people	1952
14	hate	1914
15	body	1898
16	feeling	1848
17	sleep	1816
18	hope	1794
19	worst	1779
20	ive	1766
21	heart	1755
22	night	1684
23	help	1637
24	tpain	1628
25	shit	1578
