# Supplementary material for: The Painful Tweet: Text, Sentiment, and Community Structure Analyses of Tweets Pertaining to Pain
Source: J Med Internet Res. 2015 Apr 2;17(4):e84. doi: 10.2196/jmir.3769 (PMC4400316; doi:10.2196/jmir.3769)
Supplement: Supplementary file 4 [file jmir_v17i4e84_app4.pdf]

Multimedia Appendix 4.

Appendix C. Agreement Statistics between Human Rates and Classification Methods

Classifier	Human	Sensitivity or Recall	Specificity	PPV or Precision	NPV	Accuracy	F-measure
**Rule**	**PT**	0.79	0.6	0.58	0.80	0.68	0.67
	**RG**	0.63	0.46	0.44	0.66	0.53	0.52
	**MG**	0.92	0.53	0.31	0.96	0.60	0.47
**Statistical**	**PT**	0.53	0.73	0.54	0.72	0.65	0.54
	**RG**	0.44	0.69	0.45	0.68	0.60	0.45
	**MG**	0.59	0.70	0.31	0.88	0.68	0.41
**Hybrid**	**PT**	0.56	0.73	0.56	0.73	0.66	0.56
	**RG**	0.47	0.69	0.47	0.69	0.61	0.47
	**MG**	0.65	0.70	0.33	0.89	0.69	0.44
