# Supplementary material for: The Painful Tweet: Text, Sentiment, and Community Structure Analyses of Tweets Pertaining to Pain
Source: J Med Internet Res. 2015 Apr 2;17(4):e84. doi: 10.2196/jmir.3769 (PMC4400316; doi:10.2196/jmir.3769)
Supplement: Supplementary file 5 [file jmir_v17i4e84_app5.pdf]

Multimedia Appendix 5.

Appendix D. Pain Related Tweet Volume by Hour of Day				
Hour of Day	Number of Tweets	Positive Tweets	Negative Tweets	Percent of Tweets with Positive Sentiment
0	1192	386	806	32.38
1	948	345	603	36.39
2	1548	501	1047	32.36
3	2031	608	1423	29.94
4	2452	796	1655	32.46
5	2831	996	1832	35.18
6	3608	1140	2468	31.6
7	3532	1143	2389	32.36
8	3334	1006	2328	30.17
9	3597	1155	2440	32.11
10	3179	866	2313	27.24
11	2600	703	1895	27.04
12	2855	931	1921	32.61
13	2978	888	2089	29.82
14	3243	905	2338	27.91
15	3239	948	2290	29.27
16	3488	833	2653	23.88
17	3437	925	2512	26.91
18	3765	1210	2553	32.14
19	3759	1302	2456	34.64
20	2861	900	1958	31.46
21	1786	600	1185	33.59
22	1501	447	1053	29.78
23	1226	469	757	38.25
