# Supplementary material for: The Painful Tweet: Text, Sentiment, and Community Structure Analyses of Tweets Pertaining to Pain
Source: J Med Internet Res. 2015 Apr 2;17(4):e84. doi: 10.2196/jmir.3769 (PMC4400316; doi:10.2196/jmir.3769)
Supplement: Supplementary file 6 [file jmir_v17i4e84_app6.pdf]

Multimedia Appendix 6.

Appendix E. Graph-Level Metrics.											
	Pain	#pain	Happy	Excitement	Sad	Fear	Tired	Anguish	Apple	Manchester United	Obama
Nodes	674	252	765	720	794	995	664	508	691	940	964
Edges	432	161	438	531	482	828	364	379	526	827	770
Network Diameter	2	1	1	2	1	2	2	2	2	2	2
Average Path Length	1.011	1	1	1.004	1	1.002	1.011	1.003	1.019	1.001	1.024
Density	0.001	0.003	0.001	0.001	0.001	0.001	0.001	0.001	0.001	0.001	0.001
Weakly Connected Components	252	97	328	197	315	171	301	136	179	127	229
Proportion of Weakly Connected Components to Nodes	0.37	0.38	0.43	0.27	0.40	0.17	0.45	0.27	0.26	0.14	0.24
Strongly Connected Components	672	252	765	717	792	993	663	505	691	939	963
Nodes within Giant Component	30	22	15	51	20	561	12	93	175	442	278
Edges within Giant Component	30	22	14	50	19	560	11	92	174	448	300
Percent of Nodes within Giant Component	4%	9%	2%	7%	3%	56%	2%	18%	25%	47%	29%
