# Supplementary material for: The Painful Tweet: Text, Sentiment, and Community Structure Analyses of Tweets Pertaining to Pain
Source: J Med Internet Res. 2015 Apr 2;17(4):e84. doi: 10.2196/jmir.3769 (PMC4400316; doi:10.2196/jmir.3769)
Supplement: Supplementary file 7 [file jmir_v17i4e84_app7.pdf]

Multimedia Appendix 7.

**Appendix F. Node Level Metrics**											
	**Pain**	**#pain**	**Happy**	**Excitement**	**Sad**	**Fear**	**Tired**	**Anguish**	**Apple**	**Manchester United**	**Obama**	**p value**
Number of Nodes	674	252	765	720	794	995	664	508	691	940	964	0.53
Number of Modularity Communities	252	97	328	197	315	175	301	136	179	133	238	<0.0001
Number of Modularity Communities in Proportion to Nodes	0.37	0.38	0.43	0.27	0.40	0.18	0.45	0.27	0.26	0.14	0.25	
In-Degree Centrality (Median, Range)	0 (0,29)	0 (0,21)	0 (0,14)	0 (0,50)	0 (0,19)	0 (0,526)	0 (0,11)	0 (0,91)	0 (0,174)	0 (0,391)	0 (0,72)	<0.0001
Out-Degree Centrality (Median, Range)	1 (0,3)	1 (0,3)	1 (0,5)	1 (0,2)	1 (0,2)	1 (0,2)	1 (0,2)	1 (0,20)	1 (0,18)	1 (0,7)	1 (0,20)	<0.0001
Total Degree Centrality (Median, Range)	1 (1,29)	1 (1,21)	1 (0,14)	1 (1,50)	1 (0,19)	1 (0,19)	1 (1,11)	1 (1,91)	1 (0,174)	1 (1,391)	1 (0,72)	<0.0001
