# Supplementary material for: The Painful Tweet: Text, Sentiment, and Community Structure Analyses of Tweets Pertaining to Pain
Source: J Med Internet Res. 2015 Apr 2;17(4):e84. doi: 10.2196/jmir.3769 (PMC4400316; doi:10.2196/jmir.3769)
Supplement: Supplementary file 8 [file jmir_v17i4e84_app8.pdf]

Multimedia Appendix 8.

Appendix G. Effect Sizes of In-Degree, Out-Degree, and Total Degree Centralities of ReTweet Networks						
In-Degree	Term	Comparison Term	Count	Z-Score	p-Value	r
	#pain	pain	252	0.34464	1	0.01
	Happy	pain	765	2.19175	0.1789	0.06
	Excitement	pain	720	-3.87251	0.001	0.10
	Sad	pain	794	0.21072	1	0.01
	Fear	pain	995	-9.38638	<.0001	0.23
	Tired	pain	664	1.65873	0.4763	0.05
	Anguish	pain	508	-2.4714	0.0938	0.07
	Apple	pain	691	-3.60513	0.0028	0.10
	Manchester United	pain	940	-9.16329	<.0001	0.23
	Obama	pain	964	-1.35148	0.7088	0.03
Out Degree	Term	Comparison Term	Count	Z	p-Value	r
	#pain	pain	252	-0.1525	1	0.01
	Happy	pain	765	-3.13639	0.0142	0.08
	Excitement	pain	720	3.98167	0.0006	0.11
	Sad	pain	794	-0.98231	0.9312	0.03
	Fear	pain	995	8.74294	<.0001	0.21
	Tired	pain	664	-2.95951	0.0246	0.08
	Anguish	pain	508	2.63826	0.0612	0.08
	Apple	pain	691	3.60676	0.0028	0.10
	Manchester United	pain	940	9.88725	<.0001	0.25
	Obama	pain	964	3.86785	0.001	0.10
Total Degree Centrality	Level of Untitled 35	Comparison Term	Count	Z	p-Value	r
	#pain	pain	252	0.46359	0.9997	0.02
	Happy	pain	765	-2.19355	0.1782	0.06
	Excitement	pain	720	-0.48607	0.9996	0.01
	Sad	pain	794	-1.69832	0.4482	0.04
	Fear	pain	995	-3.20496	0.0114	0.08
	Tired	pain	664	-3.75091	0.0016	0.10
	Anguish	pain	508	-0.33965	1	0.01
	Apple	pain	691	-0.57242	0.9984	0.02
	Manchester United	pain	940	0.64013	0.9961	0.02
	Obama	pain	964	5.12197	<.0001	0.13
